# Perinatal Exposure to Perfluorooctane Sulfonate Affects Glucose Metabolism in Adult Offspring

**DOI:** 10.1371/journal.pone.0087137

**Published:** 2014-01-31

**Authors:** Hin T. Wan, Yin G. Zhao, Pik Y. Leung, Chris K. C. Wong

**Affiliations:** Partner State Key Laboratory of Environmental and Biological Analysis, Croucher Institute for Environmental Sciences, Department of Biology, Hong Kong Baptist University, Hong Kong, People’s Republic of China; Hosptial Infantil Universitario Niño Jesús, CIBEROBN, Spain

## Abstract

Perfluoroalkyl acids (PFAAs) are globally present in the environment and are widely distributed in human populations and wildlife. The chemicals are ubiquitous in human body fluids and have a long serum elimination half-life. The notorious member of PFAAs, perfluorooctane sulfonate (PFOS) is prioritized as a global concerning chemical at the Stockholm Convention in 2009, due to its harmful effects in mammals and aquatic organisms. PFOS is known to affect lipid metabolism in adults and was found to be able to cross human placenta. However the effects of *in utero* exposure to the susceptibility of metabolic disorders in offspring have not yet been elucidated. In this study, pregnant CD-1 mice (F_0_) were fed with 0, 0.3 or 3 mg PFOS/kg body weight/day in corn oil by oral gavage daily throughout gestational and lactation periods. We investigated the immediate effects of perinatal exposure to PFOS on glucose metabolism in both maternal and offspring after weaning (PND 21). To determine if the perinatal exposure predisposes the risk for metabolic disorder to the offspring, weaned animals without further PFOS exposure, were fed with either standard or high-fat diet until PND 63. Fasting glucose and insulin levels were measured while HOMA-IR index and glucose AUCs were reported. Our data illustrated the first time the effects of the environmental equivalent dose of PFOS exposure on the disturbance of glucose metabolism in F_1_ pups and F_1_ adults at PND 21 and 63, respectively. Although the biological effects of PFOS on the elevated levels of fasting serum glucose and insulin levels were observed in both pups and adults of F_1_, the phenotypes of insulin resistance and glucose intolerance were only evident in the F_1_ adults. The effects were exacerbated under HFD, highlighting the synergistic action at postnatal growth on the development of metabolic disorders.

## Introduction

The incidence of metabolic diseases (i.e. obesity, diabetes and fatty liver) has become highly prevalent globally [1], [2]. Although genetic, nutrition and environmental factors have all been associated with the development of these diseases [3], [4], epidemiological and laboratory animal studies have suggested the link between pollutant exposure (i.e. dioxin, bisphenol A (BPA), pesticides, heavy metals) and the impairment of glucose homeostasis and insulin resistance [5]. The two well-known anthropogenic pollutants, dioxins and BPA have been classified as the new diabetogenic factors [6] and are believed to affect glucose homeostasis via their actions on estrogen receptors (ER-α, -β) and/or aryl hydrocarbon receptor (AhR) [3]. However the presumed mechanisms still cannot explain the wide range of metabolic perturbations reported in both epidemiological and experimental studies. In addition to ERs and AhR, the lipid sensing and regulatory receptors, peroxisome proliferator-activated receptors PPARs are known to play pivotal roles in the regulation of insulin signaling, glucose/lipid metabolism [7] and the management of metabolic homeostasis. Of special interest is the emerging global pollutants perfluoroalkyl acids (PFAAs), which have been suggested to act on PPARs to modulate energy homeostasis [3] that warrants particular attention. In fact PFAAs have been prioritized in the European research project OBELIX in 2009 as one of the risk factor in the alternation of development programming for metabolic diseases in life.

The three notorious family members of PFAAs, perfluorooctane sulfonate (PFOS), perfluorooctanoic acid (PFOA), and perfluorohexane sulfonate (PFHxS) are globally present in the environment and are widely distributed in human populations and wildlife [8]–[11]. The two most abundant PFAAs (i.e. PFOS and PFOA) are measurable in human plasma, umbilical blood, breast milk and liver [12]–[15] and have been related to developmental toxicity, immunotoxicity and hepatotoxicity in animals [10]. Due to the potential adverse health effects of PFOS, its use in industrial production was phased out in most countries in 2002, except in China where it’s still manufactured and widely used today. In 2009, PFOS was listed under Annex B of the Stockholm Convention as one of the nine new persistent organic pollutants (POPs). Chronic PFOS exposure has been reported to cause effects on animal hepatic functions [16], [17]. The major pathological manifestations include reduced body weights and loss of body fat, accompanied with increases of liver masses [10], [18]–[20]. Histological examination of liver cells revealed peroxisome proliferation and lipid accumulation. Studies of hepatic gene expression profiles in PFOA/PFOS-treated rats highlighted that the up-regulation of genes are related to the metabolism and transport of lipids [18], [20]–[23]. Accordingly it has been postulated that its mode of action is via its pleiotropic interactions with multiple members of the nuclear hormone receptors, PPARs, constitutive androstane receptor (CAR) and pregnane X receptor (PXR) [24].

Maternal transfer of PFOS across the human placenta has been reported while the data suggest the potential harmful effects of in utero exposure to PFOS on fetus [25]. However there is no toxicological information regarding the perinatal PFOS exposure to susceptibility of metabolic disorders in the offspring. Our previous study revealed that PFOS exposure induced hepatic steatosis and altered lipid metabolisms in adult mice, suggesting the exposure may lead to non-alcoholic fatty liver disease (NAFLD) [26], which is strongly associated with type II diabetes, obesity and insulin resistance [27], [28]. In this study we investigated the effects of perinatal exposure to PFOS on glucose metabolism in the offspring and demonstrated if the effects would be exacerbated under high fat diet.

## Results

### Oral Gavage Exposure to PFOS Interferes with Maternal Glucose Metabolism

**Figure 1:**
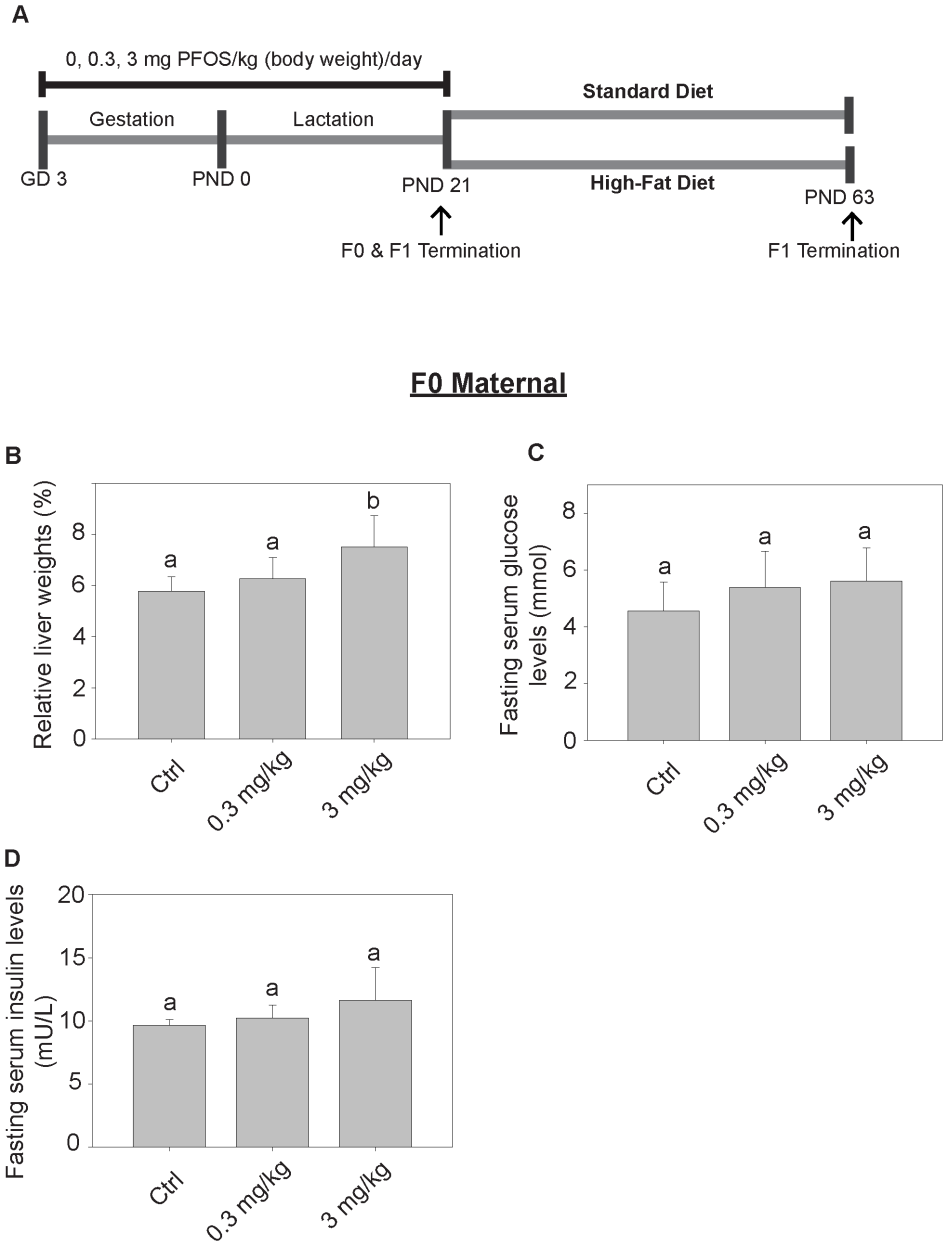
Experimental set-up and the effects of gestational and lactation PFOS exposure to maternal mice. (A) Pregnant CD-1 mice were administrated with corn oil as control, 0.3 or 3 mg PFOS/kg body weight daily by oral gavage from gestational day (GD) 3 to postnatal day (PND) 21. F_0_ maternal and some F_1_ pups were sacrificed on PND 21. The rest of the F_1_ offspring were randomly separated into two groups, allowed freely access to either standard diet or high-fat diet until termination on PND 63. The relative liver weights (B), and fasting serum glucose (C) and insulin levels (D) of maternal mice were measured (n = 6). The exposure to 3 mg PFOS/kg/day led to a significant increase in relative liver weight as compared to the control group. Bars with the same letter are not significantly different according to the results of one-way ANOVA followed by Tukey’s test (p<0.05).

Maternal mice (F_0_) were sacrificed after weaning on PND 21. PFOS concentrations in serum and liver were remarkably greater in the PFOS-exposed groups (Tables 1 and 2). The relative liver weights were significantly increased in the maternal mice of the 3 mg PFOS/kg dosed-group (Fig. 1B). The maternal body and absolute liver weights were shown (Fig. S1A–B). There is an increasing trend in both the fasting serum glucose and insulin levels towards the high-dose treated group but the data are not statistically significant (Fig/1C–D). The HOMA-IR index was calculated which measures the one’s tendency to develop insulin resistance [29]. The index was found to be significantly greater in both PFOS-treated groups as compared to the control (*p<0.02) (Table 3).

**Table 1:** The concentrations of PFOS (µg/ml) in the maternal (F_0_) serum.

Maternal (F_0_)	PFOS concentrations (µg/ml)
**Ctrl**	0.25±0.11
**0.3 mg/kg**	15.33±4.62
**3 mg/kg**	131.72±30.71

**Table 2:** The concentrations of PFOS (µg/g) in the maternal (F_0_) livers.

Maternal (F_0_)	PFOS concentrations (µg/g liver)
**Ctrl**	0.15±0.11
**0.3 mg/kg**	49.09±9.88
**3 mg/kg**	338.87±100.71

**Table 3:** Serum levels of fasting glucose-insulin and HOMA-IR index in the maternal mice.

Maternal (F_0_)	Fasting glucose (mmol)	Fasting insulin (mU/L)	HOMA-IR index
**Ctrl**	4.55±1.03	9.63±0.46	1.90±0.50
**0.3 mg/kg**	6.08±1.28	10.23±1.01	3.05±0.97*
**3 mg/kg**	5.26±1.16	11.62±2.59	3.07±0.99*

* p<0.02 as compared to the control.

### Gestation and Lactational Exposure to PFOS Affects Glucose Metabolism and Hepatic Gene Expression of the Pups at Postnatal Day 21

**Figure 2:**
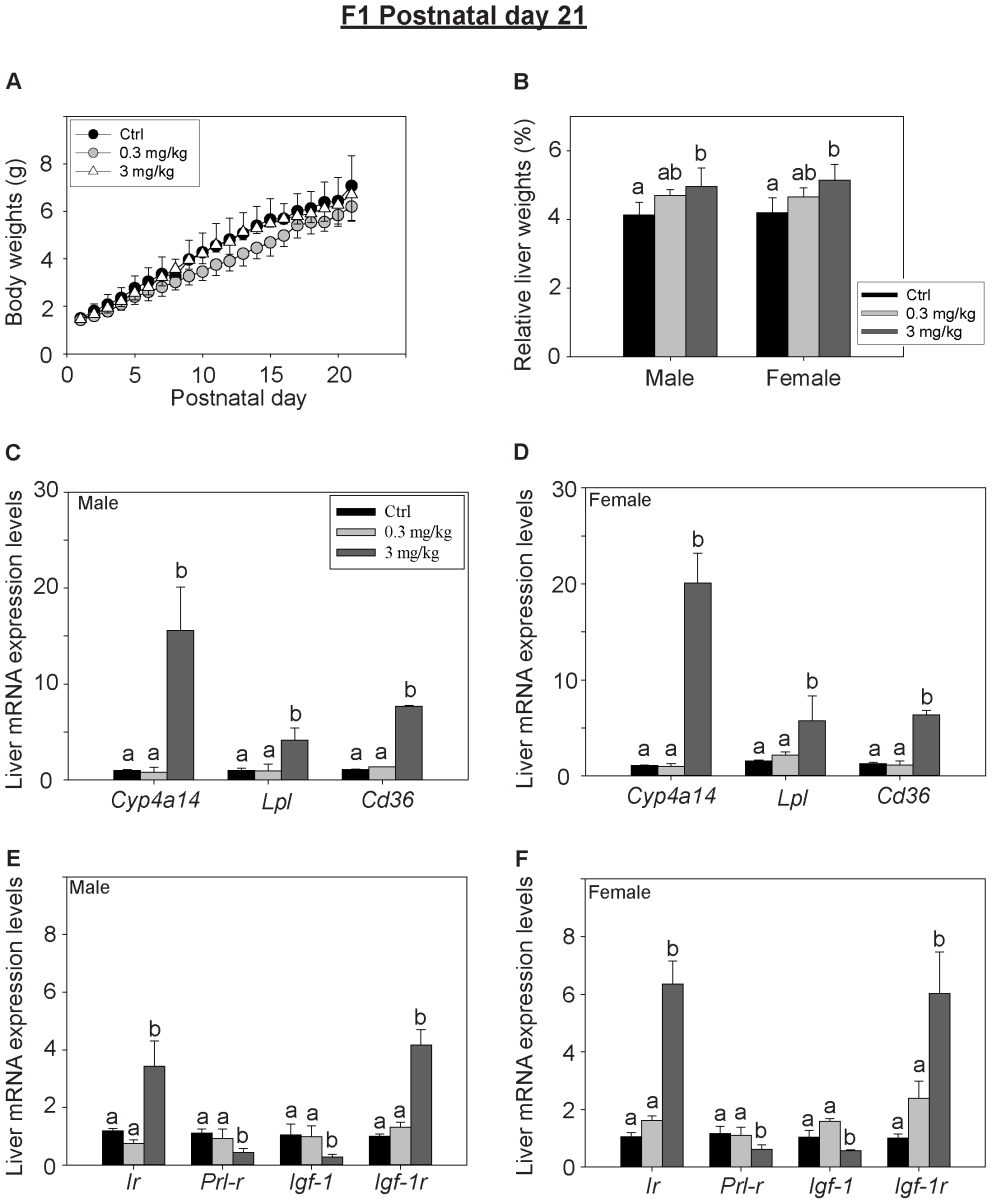
Effects of perinatal PFOS exposure on F1 pups at PND 21. (A) Body weights of the F_1_ pups were measured daily from PND 1–21. No significant differences were observed between the control and the treatment groups (n = 6 per group). (B) Liver weights of the F_1_ pups were determined at PND 21. Perinatal exposure to 3 mg PFOS/kg/day significantly increased the relative liver weights of both male and female pups as compared to the control group. Panels C–F, liver gene expression levels were determined on PND21. (C–D) Cyp4A14, lipoprotein lipase (*Lpl*) and fatty acid translocase (*Cd36*) gene expression levels were significantly up-regulated in dams of both sexes from the high-dose PFOS exposed maternal group as compared to the respective control groups. (E & F) The expression levels of the diabetic-related genes, hepatic insulin receptor (*Ir*) and insulin growth factor-1 receptor (*Igf-1r*) were significantly up-regulated in dams from the high-dose PFOS exposed groups while the expression levels of prolactin receptor (*Prl-r*) and insulin-like growth factor-1 (*Igf-1*) were decreased as compared to the respective control groups Bars with the same letter are not significantly different according to the results of one-way ANOVA followed by Tukey’s test (p<0.05).

Perinatal exposures to PFOS led to a significant accumulation of the chemical in both serum and liver of the F_1_ pups (Tables 4 and 5). The concentrations of PFOS in the liver and serum of pups are proportion to the dose of the exposure. A statistical difference between genders of pups was observed in serum PFOS contents while the level was significantly greater in the males. Although the gestation and lactational exposure did not cause noticeable effects on the body weight of the pups (Fig. 2A), the relative liver weights of perinatal PFOS-exposed pups of both sexes were significantly increased (p<0.05) (Fig. 2B). The absolute liver weights were shown in Fig. S1C. A modulation of hepatic gene expression was detected (Fig. 2C–F). The transcript levels of cytochrome P450 enzymes 4A14 (*Cyp4a14*), lipoprotein lipase (*Lpl*) and fatty acid translocase (*Cd36*) were induced in both male and female pups via perinatal exposure to 3 mg/kg PFOS (p<0.001 & 0.03 respectively) (Fig. 2C–D). The gene levels of hepatic membrane receptors were also altered. The transcript levels of insulin receptor (*Ir*) were up-regulated (p<0.001) while prolactin receptor (*Prlr*) were significantly down-regulated (p<0.03) in both sexes (Fig. 2E–F). The expression levels of the hepatic insulin-like growth factor (*Igf-1*) were significantly down-regulated while its receptor (*Igf-1r*) was up-regulated.

**Table 4:** The concentrations of PFOS (µg/ml) in the F_1_ pup serum.

	PFOS concentrations (µg/ml)	
F_1_ pups (PND 21)	Male	Female
**Ctrl**	0	0
**0.3 mg/kg**	12.73±1.96	11.35±1.08
**3 mg/kg**	98.74±4.58* (p<0.05)	87.23±4.28

* p<0.05 as compared between gender of the same treatment group.

**Table 5:** The concentrations of PFOS (µg/g) in the F_1_ pups livers.

	PFOS concentrations (µg/g liver)	
F_1_ pups (PND 21)	Male	Female
**Ctrl**	0	0
**0.3 mg/kg**	20.14±4.06	17.96±6.38
**3 mg/kg**	242.98±55.62	178.44±79.03

**Figure 3:**
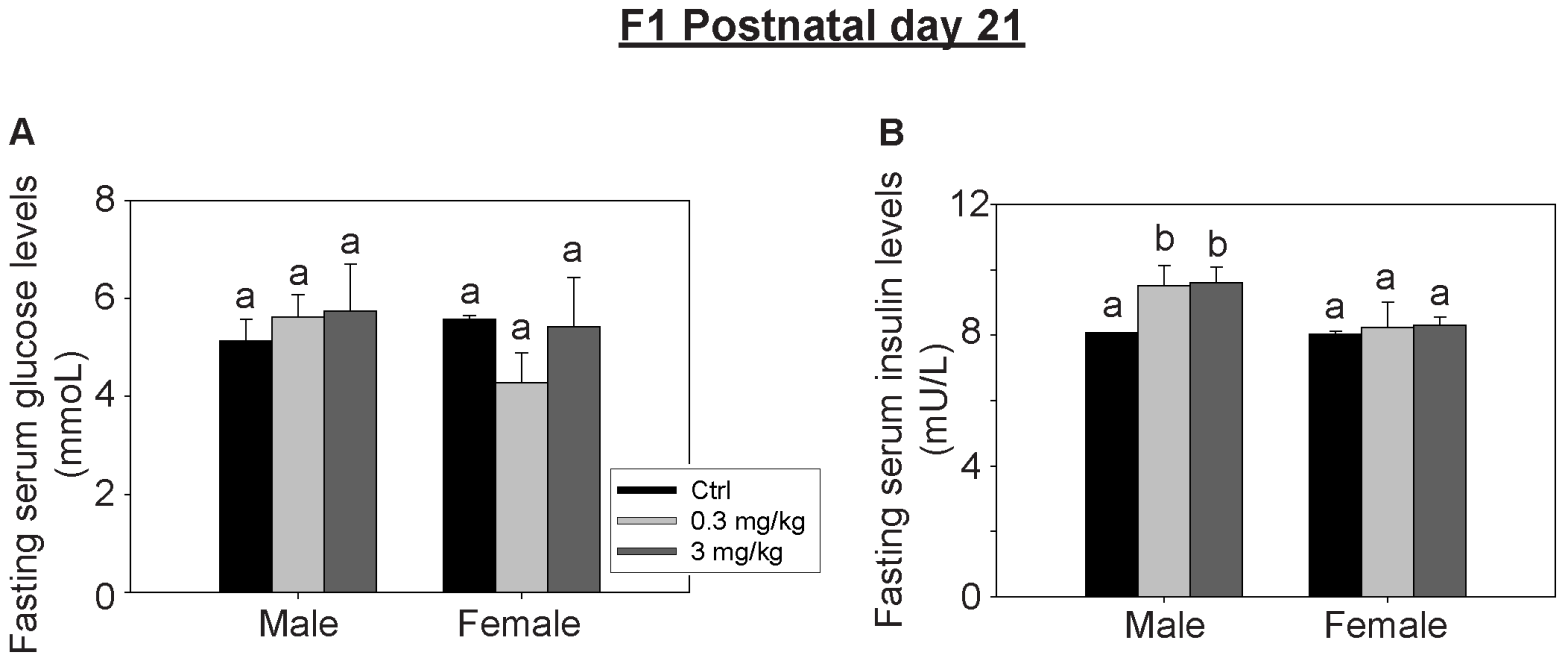
Effects of perinatal exposure to PFOS on fasting serum glucose and insulin levels of F_1_ pups at PND 21. Blood glucose and insulin levels were measured after overnight fasting (n = 6 per group). (A) Glucose levels were comparable between the treatment and control groups. (B) The insulin levels of male pups were significantly greater than the control. Bars with the same letter are not significantly different according to the results of one-way ANOVA followed by Tukey’s test (p<0.05).

To determine if the perinatal PFOS exposure disturbed glucose metabolism of the pups, serum levels of insulin and glucose were measured (Fig. 3A–B). The serum insulin levels of the perinatal PFOS-exposed male pups were significantly higher than the control, however the effects weren’t observed in the female pups. No noticeable difference in serum glucose levels between the control and perinatal PFOS-exposed groups were detected. No significant difference in the HOMA-IR index among the groups of F_1_ pups was found (Table 6).

**Table 6:** HOMA-IR index in the F_1_ pups.

	HOMA-IR index	
F_1_ pups (PND 21)	Male	Female
**Ctrl**	2.13±0.75	1.99±0.40
**0.3 mg/kg**	2.37±0.05	1.58±0.35
**3 mg/kg**	2.38±0.85	2.34±1.05

### The Effects of Perinatal PFOS Exposure on Glucose Metabolism of STD- and HFD-fed F_1_ Adults at PND 63

From PND 21 to PND 63, all weaned F_1_ mice were kept without further PFOS exposure but were fed with either standard (STD) or high-fat diet (HFD). The consumption of standard diet and high fat diet did not show significant effects on their body weight (Fig. S2). They were sacrificed on PND 63 and the body levels of PFOS were measured (Tables 7 and 8). As compared to the F_1_ pup’s data at PND 21, notable reduction of PFOS levels from serum and liver in both STD- and HFD-fed F_1_ adults were observed. Interestingly HFD-fed F_1_ adults accumulated significantly greater levels of serum and liver PFOS than the STD-fed F_1_ adults (P<0.05), indicating that the consumption of HFD led to a slower elimination rate of PFOS.

**Table 7:** The concentrations of PFOS (µg/ml) in the F_1_ adult serum.

	PFOS concentrations (µg/ml)			
F_1_ adults (PND 63)	Male		Female	
	STD	HFD	STD	HFD
**Ctrl**	0	0	0	0
**0.3 mg/kg**	0.30±0.06	1.20±0.29*	0.51±0.11	1.50±0.27*
**3 mg/kg**	3.36±1.07	5.38±0.30*	3.40±1.08	5.76±1.24*

* p<0.05 as compared between STD and HFD diets under the same gender groups.

**Table 8:** The concentrations of PFOS (µg/g) in the F_1_ adult livers.

	PFOS concentrations (µg/g liver)			
F_1_ adults (PND 63)	Male		Female	
	STD	HFD	STD	HFD
**Ctrl**	0	0	0	0
**0.3 mg/kg**	3.97±0.50	5.43±0.98*	3.34±0.50	4.27±1.75*
**3 mg/kg**	12.30±1.59	24.54±1.06*	13.77±4.05	21.34±3.36*

* p<0.05 as compared between STD and HFD diets under the same gender groups.

**Figure 4:**
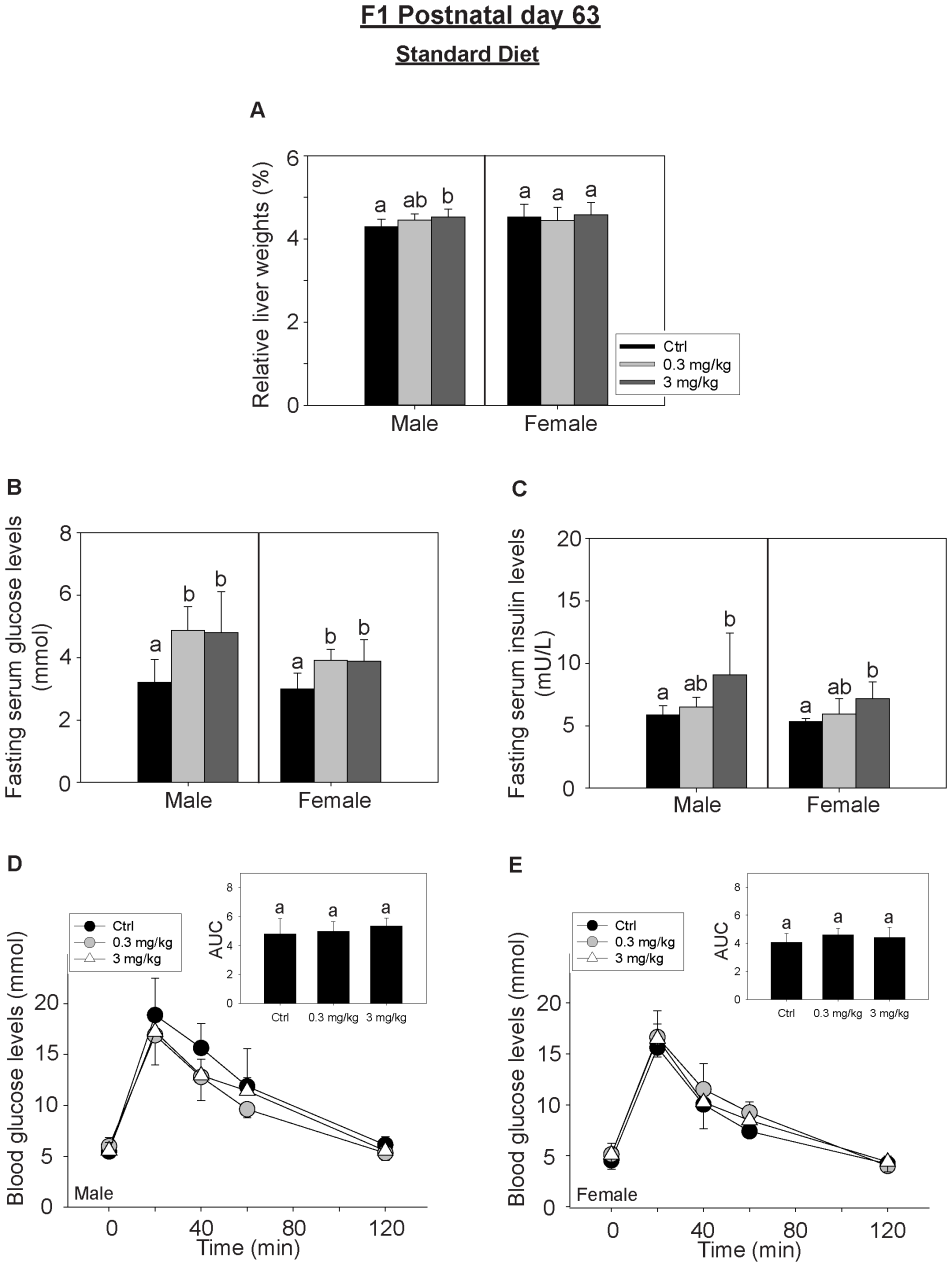
The effects perinatal PFOS exposure to STD-fed F_1_ adult offspring at PND63. F_1_ offspring were fed with the standard diet (STD) and grown without further PFOS exposure. The F_1_ adults were sacrificed on PND 63. The relative liver weights, fasting blood glucose and insulin levels were measured and OGTT was performed. (A) The relative liver weights of the male adults (F_1_) from the high-dose maternal group were significantly increased (n = 8) as compared to the control group. (B) Fasting blood glucose levels were increased in the F_1_ adults of both sexes from PFOS-exposed maternal groups (n = 6) as compared to the respective control groups. (C) Fasting blood insulin levels in the F_1_ adults of both sexes from the high-dosed exposed group were noticeably increased (n = 6) as compared to the respective control groups. In panels D–E, F_1_ adults were given 2 g glucose/kg body weight by oral gavage at time 0 (min) and blood glucose levels were measured by the glucometer at specific time intervals. The blood glucose levels reached maximum at time 20 (min) and gradually drop to baseline levels at time 120 (min). OGTT results of the F_1_ male (D) and female adults (E) were shown (n = 4). The data at the same time point from the control and the perinatal PFOS treated groups were compared. No statistical differences were detected. The area under curve (AUC) analysis showed that there were no significant differences between the respective control and the treatment groups. Bars with the same letter are not significantly different according to the results of one-way ANOVA followed by Tukey’s test (p<0.05).

In the STD-fed group, the relative liver weights of male pups from high-dosed maternal group were significantly higher than the control pups (Fig. 4A). The absolute liver weights of the pups were also shown in Fig. S1D. The serum fasting glucose and insulin levels of pups of both sexes from PFOS-maternal groups were significantly higher than pups from the control-maternal group (Fig. 4B–C). OGTT was carried out to investigate the dynamic changes of glucose elimination in the STD-fed F_1_ adults (Fig. 4D–E). No significant differences were observed as compared to the control F_1_ adults.

**Figure 5:**
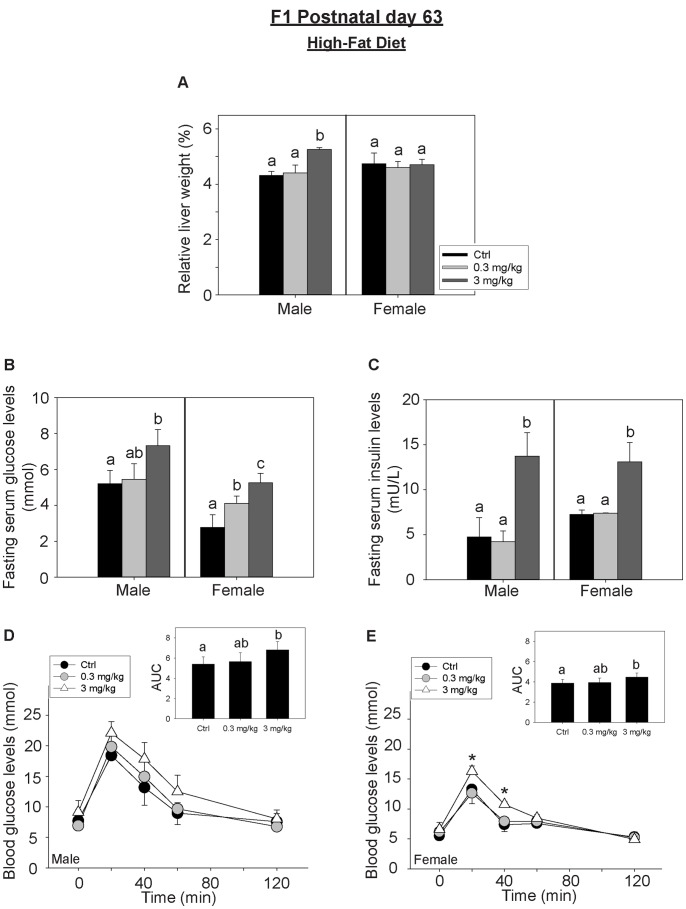
The effects perinatal PFOS exposure to HFD-fed F_1_ adult offspring at PND63. F_1_ offspring were fed with the high-fat diet (HFD) and grown without further PFOS exposure. The F_1_ adults were sacrificed on PND 63. The relative liver weights, fasting blood glucose and insulin levels were measured and OGTT was performed. (A) The relative liver weights of the male adults (F_1_) from the high-dose maternal group were significantly increased (n = 8) as compared to the control group. (B) Fasting blood glucose levels were increased in the F_1_ adults of both sexes from PFOS-exposed maternal groups (n = 6) as compared to the respective control groups. (C) Fasting blood insulin levels in the F_1_ adults of both sexes from the high-dosed exposed group were noticeably increased (n = 6) as compared to the respective control groups. The data of the OGTT from the F_1_ male (D) and female adults (E) were shown. The data at the same time point from the control and the perinatal PFOS treated groups were compared. The area under curve (AUC) analysis showed that there were significant increases in the F_1_ adults of both sexes as compared to the respective control groups. Bars with the same letter are not significantly different according to the results of one-way ANOVA followed by Tukey’s test (p<0.05).

In the HFD-fed group, a significant increase in the relative liver weights, fasting serum glucose and insulin levels of the male F_1_ adults from high-dosed PFOS maternal groups was observed (Fig. 5A–C). The absolute liver weights were shown in figure S1E. Significant effects on fasting glucose and insulin levels were measured in the perinatal exposed female F_1_ adults. OGTT experiments demonstrated that the blood glucose area under the curve (AUC) was significantly increased in the HFD-fed F_1_ adults of both sexes from the high-dosed PFOS maternal group (*p<0.02) (Fig. 5D–E). Comparisons of the effects of the STD and HFD on perinatal PFOS-exposed F_1_ adult offspring at PND63 were shown (Fig. S3). The relative liver weights and fasting blood glucose levels of the HFD-fed male adult offspring from the high-dose maternal group were significantly higher than that in the respective group in the STD. While the fasting blood insulin levels in the HFD-fed female adults (F_1_) from the high-dosed exposed group were noticeably increased as compared to the respective group in the STD. Table 9 shows comparisons of HOMA-IR index among STD-/HFD-fed F_1_ adults of both sexes. The index in HFD-fed F_1_ adults was noticeably greater than the respective STD-fed pups. In the HFD-fed F_1_ adults, the influence of perinatal PFOS exposure to HOMA-IR index was more prominent in the males than the females (P<0.001 for perinatal low-dose, P<0.05 for perinatal high-dose). In addition to the OGTT, insulin tolerance tests were performed. However no significant differences in glucose responses were observed among the control and the treatment groups (Fig. S4).

**Table 9:** The HOMA-IR index in STD- and HFD-fed F_1_ adults.

	HOMA-IR index			
F_1_ adults (PND 63)	Male		Female	
	STD	HFD	STD	HFD
**Ctrl**	0.99±0.52	1.11±0.41	0.71±0.13	0.93±0.16
**0.3 mg/kg**	1.40±0.23	3.01±0.33#	1.02±0.17	1.46±0.13#
**3 mg/kg**	1.90±0.76*	5.01±2.22*#	1.17±0.43*	2.99±1.40*#

* p<0.01 as compared with the respective control groups.

# p<0.01 as compared between STD and HFD diets under the same gender groups.

## Discussion

The notorious member of PFAAs, perfluorooctane sulfonate (PFOS) is prioritized as a global concerning chemical. Although industrial production of PFOS in most countries has already been ended since 2002, its contamination is still reported in our living environment and human blood samples, implying the exposure risk of our population to this compound. Since a number of reports have demonstrated PFOS-elicited metabolic syndromes in adult animals, we are interested in deciphering if perinatal PFOS exposure would increase susceptibility of metabolic diseases in the offspring. In this study, perinatal exposure to PFOS was performed to investigate the immediate and chronic effects of PFOS exposure on glucose metabolisms in offspring. In considering the uncertainty factors encountered in animal experiments, the oral gavage doses for maternal mice (F_0_) were set at 0.3 to 3 mg/kg day, which is 10 to 100-fold higher than the human equivalent dose of exposure for general public but is comparable to the occupational exposure levels [30]. According to our data, the PFOS exposure dose to fetal and weaning pups (F_1_), those indirectly receiving 6.2–10.7% of the maternal body load via placenta and lactation, would be equivalent to the exposure levels for general public. Our data illustrated the first time the effects of the environmental equivalent dose of PFOS exposure on the disturbance of glucose metabolisms in F_1_ pups and adults at PND 21 and 63 respectively. Although the biological effects of PFOS on the elevated levels of fasting serum glucose and insulin levels were observed in both pups and adults of F_1_, the phenotypes of insulin resistance and glucose intolerance were evident (i.e. HOMA-IR index and glucose AUC) in the F_1_ adults. The effects were exacerbated under HFD, highlighting the synergistic action of chemical stressor and nutrition on the development of metabolic disorders.

### Effects of PFOS Exposure on Glucose Metabolism in Maternal Mice

Similar to our previous findings, oral gavage exposure of maternal mice to PFOS caused significant increases in relative liver weights [26]. To relate the body compartment distribution of PFOS in liver and blood, an approximate 3∶1 ratio was observed [10]. The remaining PFOS were probably partitioned in other body fluids (i.e. milk), tissues/organs, conceptus or excreted [31]. PFOS was known to bind with proteins [i.e. human serum albumin (HSA)] by interactions between the polar sulphonic groups to the hydrophilic residue of HSA to form a compact structure [32]. This structural interaction renders the relatively long human serum half-life (5.4 years) among other PFCs and chemical pollutants [8]. A previous toxicokinetic report demonstrated that the body elimination half-life of PFOS in rodents was about 1–2 months [33]. In contrast to the 5.4 years of the PFOS half-life in humans, it seems that the rodents displayed a relatively higher serum elimination rate.

Biochemical analysis of glucose metabolism illustrated that the chronic exposure had no noticeable effects on homeostatic regulation of blood glucose and insulin in the PFOS-exposed maternal mice as compared to the control. However the calculated HOMA-IR index was found to be significantly greater in the high-dose maternal group. The HOMA-IR index is known to be a clinical parameter to evaluate hepatic insulin sensitivity and is useful to predict the risk of hypertension and type II diabetes in human diagnostics [34], [35]. Therefore the observation indicated that PFOS exposure may be a potential chemical stressor to disrupt glucose metabolism during pregnancy.

### Effects of Maternal Transfer and Lactational Exposure to PFOS on F_1_ Pups at PND 21

It is known that PFOS can cross placenta and through lactation to impose developmental effects on fetus and neonates [25], [31], [36]–[40]. To investigate if the increase of liver weight might cause disturbance to liver functions as reported in the toxicological models in adult animals [22], [23], [26], [41], [42], the mRNA expression levels of hepatic genes targeting lipid metabolism were measured in the F_1_ pups and were found to be modulated. Although the data showed perturbations of the gene expressions in related to lipid metabolism, further work are needed to elucidate if the changes at the transcript levels are linked with protein changes and so an alteration in liver function.

Recent studies on the perinatal exposure to BPA or phthalates (i.e. DEHP) were reported to illustrate negative impacts of the exposure on the regulation of blood glucose and insulin in rodents [43]–[45]. However data of metabolic effects of PFOS intoxication to fetal and neonates are largely not known. In this study, the measurement of fasting serum levels of glucose and insulin (indices of insulin resistance) indicated that the male pups from PFOS-exposed maternal groups showed significantly greater serum insulin levels than the control pups. A modulation of hepatic gene expression in glucose metabolism was observed in the perinatal PFOS-exposed pups. The transcript level of the hepatic insulin receptor (*Ir*) was found to be remarkably up-regulated while the prolactin receptor (Prlr) was down-regulated. The hepatic IGF-1/IGF-1r axis was also disrupted in the perinatal PFOS-exposed pups. Prolactin [46] and insulin are diabetic-related hormones. An alternation of their receptor gene expression levels suggested a modulation of their respective functions in glucose metabolism. The upregulation of *Ir* transcripts might reflect physiological changes to insulin responsiveness. Supposedly if the gene effects were recapitulated at the protein levels, the HOMA-IR would be lower. However our data showed that significant higher HOMA-IR indexes in the perinatal PFOS-exposed adult offspring were measured. It suggests that the *Ir* gene expression might not be linked to the protein level. Moreover PFOS-elicited effects might affect insulin receptor-signaling and so the increase in Ir expression couldn’t rescue the effects of the perturbations. The pro-insulin factor, IGF-1 is known to confer insulin-like action to stimulate glucose uptake and its mal-regulation is recognized to associate with insulin resistance and diabetes [47]. The up-regulation of hepatic IGF-1 receptor in perinatal PFOS-exposed pups may be due to the negative feedback from low level of hepatic IGF-1 biosynthesis. Patients with obesity, metabolic syndrome and diabetes showed significant lower plasma levels of IGF-1 [48]. The consistent metabolic effects demonstrated in both sexes of the perinatal PFOS-exposed pups rendered us to determine the long-term effects of the exposure on insulin resistance and glucose intolerance in the F_1_ adult offspring.

### Effects of Perinatal PFOS Exposure on Glucose Metabolism in F_1_ Adult Offspring at PND 63

From PND 21 to 63, there were about 32–78% and 14–55% reductions in hepatic PFOS levels, respectively in the STD- and HFD-fed F_1_ adults. The PFOS elimination rate was greater in the F_1_ adults fed with the STD than the HFD. This observation indicates that dietary fat contents would modulate PFOS elimination from the animals. Although there is no study to indicate toxicokinetic difference in the elimination PFOS or related compounds, other study using a non-steroidal anti-inflammatory drug suggested that high dietary fat contents increased the half-life elimination of the drug in beagle’s livers, owing to an alteration in the expression levels of efflux transporters [49]. The mechanism of HFD consumption in affecting PFOS toxicity is largely unknown, the possible effects on xenobiotic efflux transporters warrants further investigation. Male F_1_ adults in general eliminated less and/or accumulated higher levels of PFOS in their livers. In the STD-fed F_1_ adults, the ranges of hepatic PFOS accumulation in males (4.78–16.59 µg/liver) and females (3.48–14.91 µg/liver) while in the HFD-fed F_1_ adults, the levels were ranging from (6.45–37.2 µg/liver) in the males and (4.15–28.75 µg/liver) females. The underlying mechanisms of the gender-specific metabolism of PFOS is not known, however it is generally known that most drugs are cleared faster in females than males [50]. It may be probably due to sexual-dimorphic variations in the induction of hepatic cytochrome P450s, regulated by the pregnane X-receptor (PXR) and constitutive androstane receptor (CAR) in drug metabolism [51]–[53].

Both STD- and HFD-fed F_1_ adults from PFOS-exposed maternal groups showed the elevated levels of fasting serum glucose and insulin as compared to the respective control groups. Consistently a significant greater in HOMA-IR index was observed in both STD- and HFD-fed PFOS-exposed F_1_ adults. The data suggested the negative effects of the perinatal PFOS exposure to glucose metabolism in the F_1_ adults. Moreover the significant greater values of HOMA-IR index in HFD-fed F_1_ adults than the STD-fed groups underlined the synergistic effects of HFD on the development of insulin resistance in the animals. The synergistic effects were also exemplified in the oral glucose tolerance test (OGTT), while the glucose AUCs in HFD-fed F_1_ adults were significantly greater than the STD-fed groups. Remarkably a human epidemiological study has demonstrated that serum levels of PFOS are associated with elevated serum insulin, HOMA-IR and altered β-cells functions [54]. Other than PFOS, in utero exposure to some toxicants such as arsenic, nicotine, organotins, phthalates, BPA, and pesticides were also shown to have positive association to the development of obesity, type II diabetes and insulin resistance [55]. Moreover nutrition is one of the non-chemical stressors which may contribute to disease progression in animals exposed to chemical toxicants [56]. The consumption of high-calorie, high-fat diet is one of the well-known risk factors to metabolic diseases [57]. Both nutritional and chemical stresses may exacerbate disease-associated biochemical factors in animals, such as induction of oxidative stress and inflammatory cytokines, to promote metabolic dysfunction in livers. Herein, we demonstrated that HFD can increase the susceptibility of perinatal PFOS-exposed adult offspring to metabolic disorders.

Increasing research studies have focused on the effects of perinatal exposure to determine if chemical pollutants predispose offspring to various kinds of metabolic disorders, underlining the risk of the exposure via maternal transfer. Our study indicated that perinatal exposure to PFOS caused disturbance to glucose metabolism in pups at PND 21. The development of insulin resistance and glucose intolerance was evident in adult offspring at PND 63. Further investigation of the effects on fetal liver and pancreas development would warrant a better understand of the underlying mechanistic actions of PFOS-induced metabolic disorders.

## Materials and Methods

### Experimental Animals and Chemicals

All experimental animals were housed and handled in accordance with the Guidelines and Regulations of Department of Health, the Government of Hong Kong Special Administrative Region. The protocol was approved by the Committee on the Use of Human and Animal Subjects of the Hong Kong Baptist University (Permit no. 261812). Female CD-1 mice (6–8 week old) were purchased from Laboratory Animal Service Centre of the Chinese University of Hong Kong (Hong Kong, China). The entire study was repeated for four times with mice that were received in separate batches. The animals were acclimatized for 1 week before experiments. Mice were allowed to mate for two consecutive nights and were randomly divided into three groups (about 6 individuals per group). Each group was housed in polypropylene cages with sterilized bedding and was maintained under controlled temperature (22**°**C) and 12L:12D cycles (0600–1800 h). The mice were weighted by an electronic balance (Shiamdzu, Tokyo, Japan) and orally administered 0.3 and 3 mg PFOS/kg body weight by gavage in corn oil in the afternoon from the last day of mating, then daily throughout gestation until the end of weaning period (PND 21) (Fig. 1A). Perfluorooctane sulfonate (98% purity, Sigma-Aldrich, US) was dissolved in dimethyl sulfoxide (DMSO, Sigma-Aldrich, US) before mixing with corn oil (the final concentration of DMSO is less than 0.05% in all group). The control group was given corn oil with 0.05% DMSO. The F_0_ maternal mice were fed with standard food (Rodent Diet 5001; LabDiet) and water *ad libitum*. After weaning, 2 pups per dam and all F_0_ mice were sacrificed at postnatal day 21 for follow-up experiments. The rest of the pups were randomly divided into two groups, and were fed with either standard diet (STD) (Rodent Diet 5001; LabDiet) or high fat diet (HFD) (MP Biomedicals, US) and were sacrificed at PND63.

All the mice were fasted overnight (16 h) and killed by cervical dislocation in the morning on the designed dates. Blood sample was collected by cardiocenthesis, and serum was prepared by centrifugation at 3000×g for 15 min. The sera were stored at −20**°**C immediately until further analysis.

### Liver and Blood Serum PFOS Analysis

A mass-labeled standard solution for PFOS (used as the internal standard) was purchased from Wellington Laboratories (Ontario, Canada). Purities of the analytical standard were greater than 98%. The method for the extraction and analysis of PFOS was performed as previously described [58]. Briefly, liver sample was homogenized in MilliQ water, while serum sample was diluted in 1 ml of MilliQ water (n = 4 for each group per experiment). One ml of liver homogenate or the diluted serum sample was then mixed with 1 ml tetra-n-butyl ammonium hydrogen sulfate (TBA), 2 ml TBA buffer and 5 ml methyl-tert-butyl ether (MTBE), followed by shaking for 30 min at 300 mot/min at room temperature. After centrifugation at 3,500 rpm for 15 min, the supernatant (organic phase) was transferred to a clean 50 ml polypropylene tube. The remaining aqueous phase was subjected to extraction twice with 5 ml MTBE. All three organic phases were pooled and were concentrated to dryness under a gentle stream of nitrogen and reconstituted with 1 mL of 10 mM Ammonium/Acetate:Acetonitrile (6∶4) prior to LC/MS/MS analysis. Standards of PFOS and labeled-PFOS used for calibration were both prepared in methanol. The detection of PFOS was performed using an Agilent 1200 high-performance liquid chromatograph coupled with tandem mass spectrometry (HPLC-MS/MS, Aglient 1200 series, Aglient Technologies, California, US). A 30 µL aliquot of the extract was injected into a guard column (Zobrax Eclipse Plus-C8, 2.1 mm i.d. ×12.5 mm length, 5-µm; Agilent Technologies), which was connected to a Zorbax Eclipse Plus C8 column (2.1 mm i.d. x100 mm length, 3.5-µm; Agilent Technologies). Instrumental parameters for analysis were described in Zhao and coworkers [59]. LOD was defined as 3-fold higher than the signal-to-noise ratio and 0.4 ng/ml for PFOS. LOQ was defined as 10-fold higher than the signal-to-noise ratio. The vales of matrix recovery were 99.95%.

### RNA Isolation and Real-Time PCR

RNA isolation was carried out by TriReagent according to manufacturer’s instructions. Total RNA with A260:A280 ratio above 1.85 was used for real-time PCR analyses. Complementary DNA was synthesized from 150 ng of total cellular RNA using High Capacity RNA-to-cDNA Master Mix (Applied Biosystem, Foster City, CA). Gene-specific primers were designed from published sequences (Table S1). Real-time PCR was conducted with a program of 3 min at 95°C followed by 40 cycles of 95°C for 15 sec, 56°C for 20 sec, and 72°C for 30 s. Standards and cDNAs from samples were quantified using StepOne Real-Time PCR system using SYBR Green Master mix (Applied Biosystems). By applying the comparative CT method [60], the data were presented as relative to the mouse actin and normalized to the control. The error bars in the control group displayed the variation of different mice within the group. Statistical analysis was performed using normalized data by Sigma Stat (version 3.5) while simple t-test analysis was conducted. Occurrences of primer-dimers and secondary products were evaluated using melting curve analysis. Control amplifications were done either without RT or without RNA. All glassware and plastic ware were treated with diethyl pyrocarbonate and autoclaved.

### Fasting Serum Levels of Glucose and Insulin

Serum fasting glucose (n = 6 per group) was measured by StanBio Glucose Liquicolor (StanBio Laboratory, Boerne, US) according the manufacturer’s manual. Serum insulin (n = 6 per group) was determined by a mouse insulin ELISA kit (Mercodia, Sweden) according to manufacturer’s protocol. A Homeostatic Model Assessment for Insulin Resistance (HOMA-IR index) was calculated by blood glucose (mmol/L) × insulin (mU/L)/22.5 [29].

### Oral Glucose Tolerance Test (OGTT)

Mice were fasted for 16 h before the experiment (n = 4 per group). The fasting glucose level was measured by Accu-chek Glucometer (Roche, US). After the measurement, 2 g/kg body weight glucose solution was given to the mice orally by gavage and blood glucose was measured at time 15, 30, 60 and 120 min. Glucose response during the OGTT was calculated by the area under the curve (AUC) using the trapezoidal method [61].

### Intraperitoneal Insulin Tolerance Test (ITT)

Mice were fasted for 16 h before the experiment (n = 4 per group). The fasting glucose level was measured by Accu-chek Glucometer (Roche, US). After the initial measurement, intraperitoneal injections of insulin (0.5 U/kg body weight, Sigma) were given to the mice and blood glucose levels were measured at time 15, 30, 45 and 60 min.

### Statistical Analysis

Statistical evaluations were conducted by SigmaStat 3.5. All data were tested to be normally distributed and independent with significance of 0.05. Differences between treatment groups and corresponding control groups were tested for statistical significance by one-way ANOVA followed by Tukey’s test (significance at p<0.05) or Student’s *t*-tests as appropriate. Results are presented as the mean ± SD.

## Supporting Information

Figure S1 **Pregnant CD-1 mice were administrated with corn oil as control, 0.3 or 3 mg PFOS/kg body weight daily by oral gavage from gestational day (GD) 3 to postnatal day (PND) 21.** F_0_ maternal (n = 6 per group) were sacrificed on PND 21. The body weight (A) and absolute liver weights (B) were measured. No significant differences were observed between the control and the treatment groups. The absolute liver weights of F_1_ pups on PND 21 (C) and PND 63 (D–E) were shown. Bars with the same letter are not significantly different according to the results of one-way ANOVA followed by Tukey’s test (p<0.05).
(TIFF)

Figure S2 **F_1_ adult offspring were fed with either the standard diet (STD) or high fat diet (HFD) after weaning (PND 21).** The body weights were measured weekly (n = 11 per each group). No significant differences were observed between the control and the perinatal PFOS exposed groups from either the STD or HFD groups.
(TIFF)

Figure S3 **Comparisons of the effects of STD and HFD on perinatal PFOS-exposed F_1_ adult offspring at PND63.** F_1_ offspring were fed with either standard diet (STD) or high fat diet (HFD) and grown without further PFOS exposure. The relative liver weights, fasting blood glucose and insulin levels of the STD- and HFD-fed F_1_ were shown. The relative liver weights (n = 8) (A) and fasting blood glucose levels (n = 6) (C) of the HFD-fed male adults (F_1_) from the high-dose maternal group were significantly higher than that in the respective group in the STD (^#^p<0.05, student’s t test). (F) Fasting blood insulin levels in the HFD-fed female adults (F_1_) from the high-dosed exposed group were noticeably increased as compared to the respective group in the STD (n = 6, ^#^p<0.05, Student’s t test). For the comparison of the control and treatment groups under either the STD or HFD, bars with the same letter are not significantly different according to the results of one-way ANOVA followed by Tukey’s test (p<0.05).
(TIFF)

Figure S4 **Effects of perinatal PFOS exposure on glucose responses in the insulin tolerance test (ITT).** F_1_ adult offspring were given intraperitoneal injection of insulin (0.5 U insulin/kg body weight) at time 0 (min). Blood glucose levels were measured by the glucometer at the designated time intervals (15, 30, 45 and 60 min). The ITT data of the adult offspring from the STD (A & B) and HFD (C & D) were shown (n = 4). The data at the same time point from the control and the perinatal PFOS treated groups were compared using one-way ANOVA (p<0.05). Statistical analysis showed that there were no significant differences among the control and the perinatal PFOS-exposed groups.
(TIFF)

Table S1 **Nucleotide sequences of primers used in the present study.**
(DOC)
