# Supplementary figures and images for: Perinatal Exposure to Perfluorooctane Sulfonate Affects Glucose Metabolism in Adult Offspring
Source: PLoS One. 2014 Jan 31;9(1):e87137. doi: 10.1371/journal.pone.0087137 (PMC3909066; doi:10.1371/journal.pone.0087137)

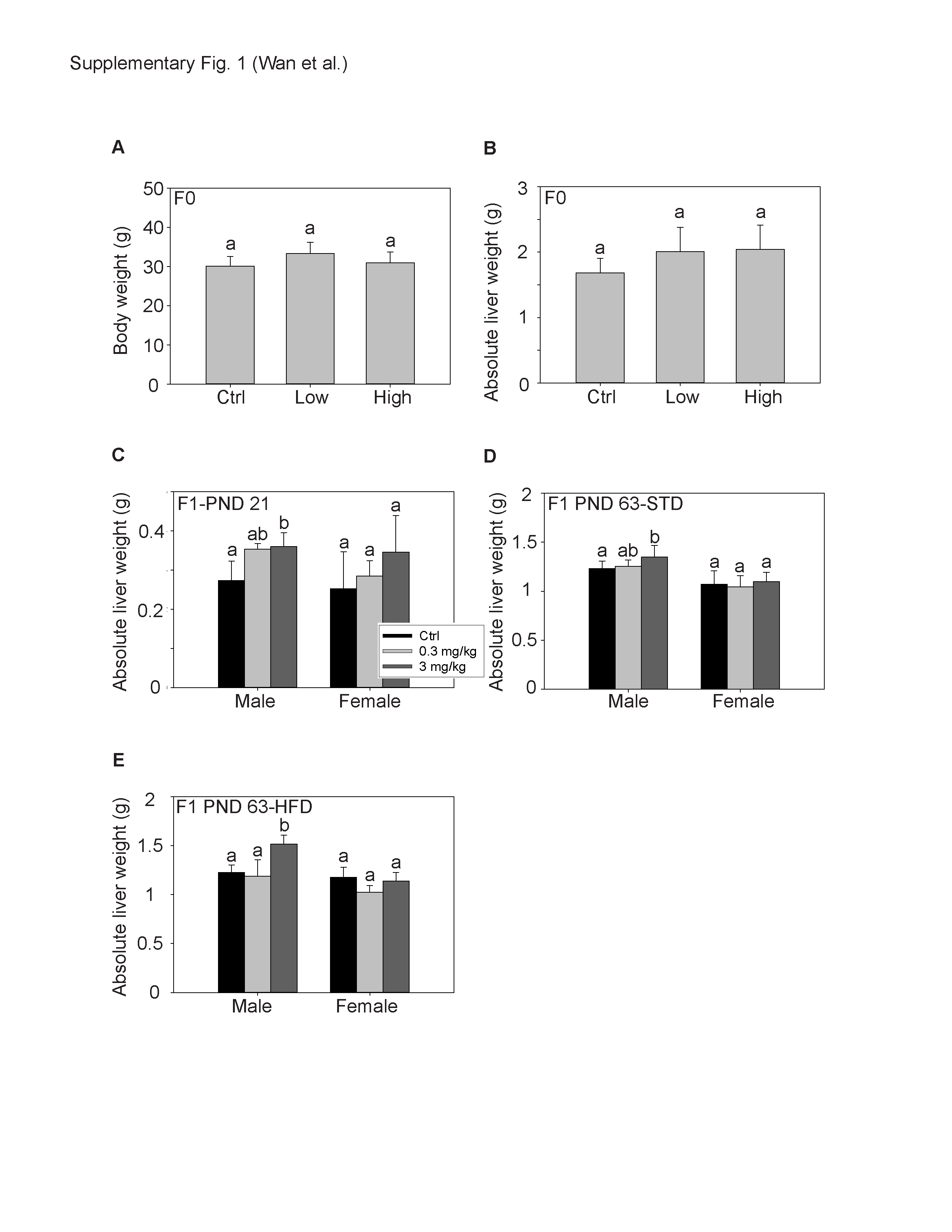

Supplement: Figure S1 — Pregnant CD-1 mice were administrated with corn oil as control, 0.3 or 3 mg PFOS/kg body weight daily by oral gavage from gestational day (GD) 3 to postnatal day (PND) 21. F0 maternal (n = 6 per group) were sacrificed on PND 21. The body weight (A) and absolute liver weights (B) were measured. No significant differences were observed between the control and the treatment groups. The absolute liver weights of F1 pups on PND 21 (C) and PND 63 (D–E) were shown. Bars with the same letter are not significantly different according to the results of one-way ANOVA followed by Tukey’s test (p<0.05). (TIFF) [file pone.0087137.s001.tiff]

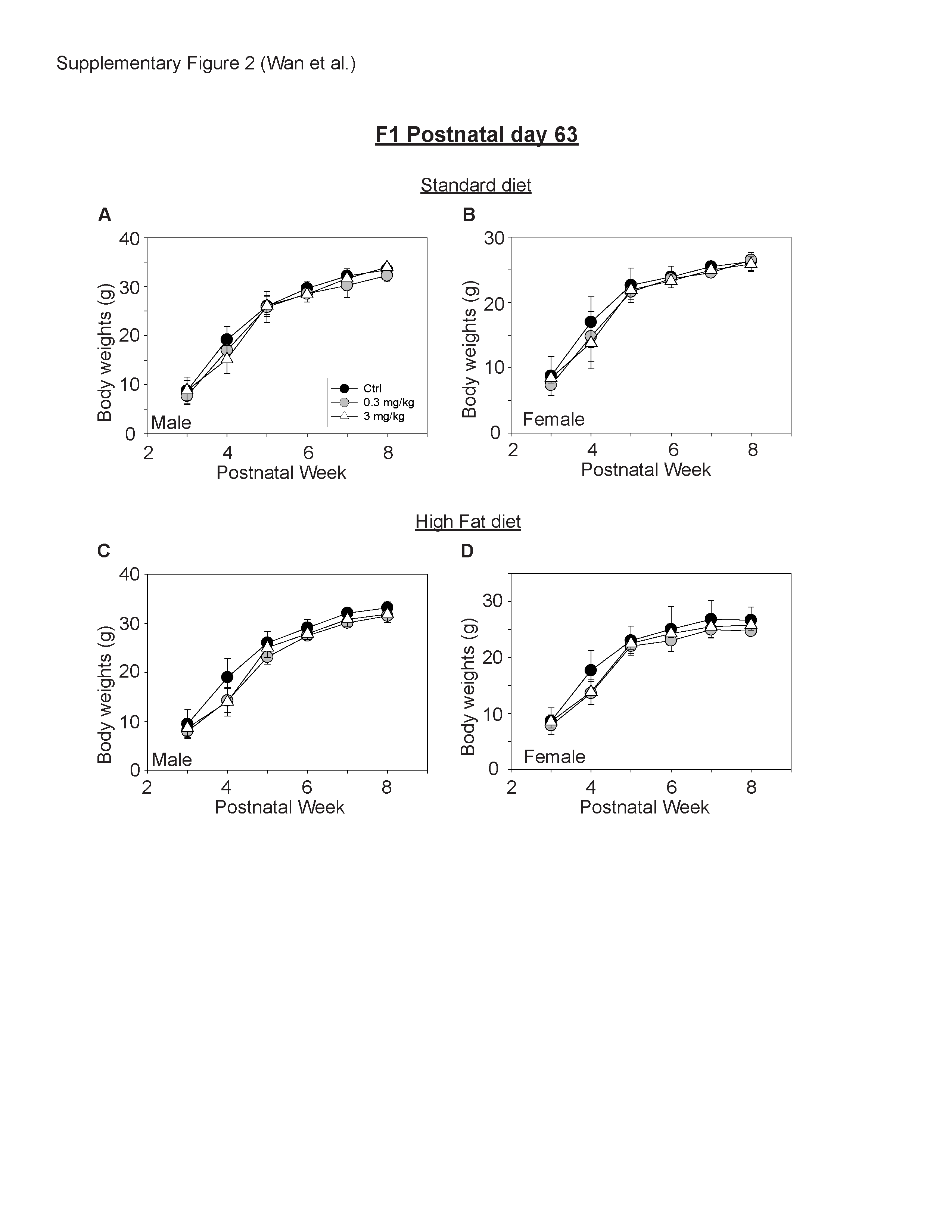

Supplement: Figure S2 — F1 adult offspring were fed with either the standard diet (STD) or high fat diet (HFD) after weaning (PND 21). The body weights were measured weekly (n = 11 per each group). No significant differences were observed between the control and the perinatal PFOS exposed groups from either the STD or HFD groups. (TIFF) [file pone.0087137.s002.tiff]

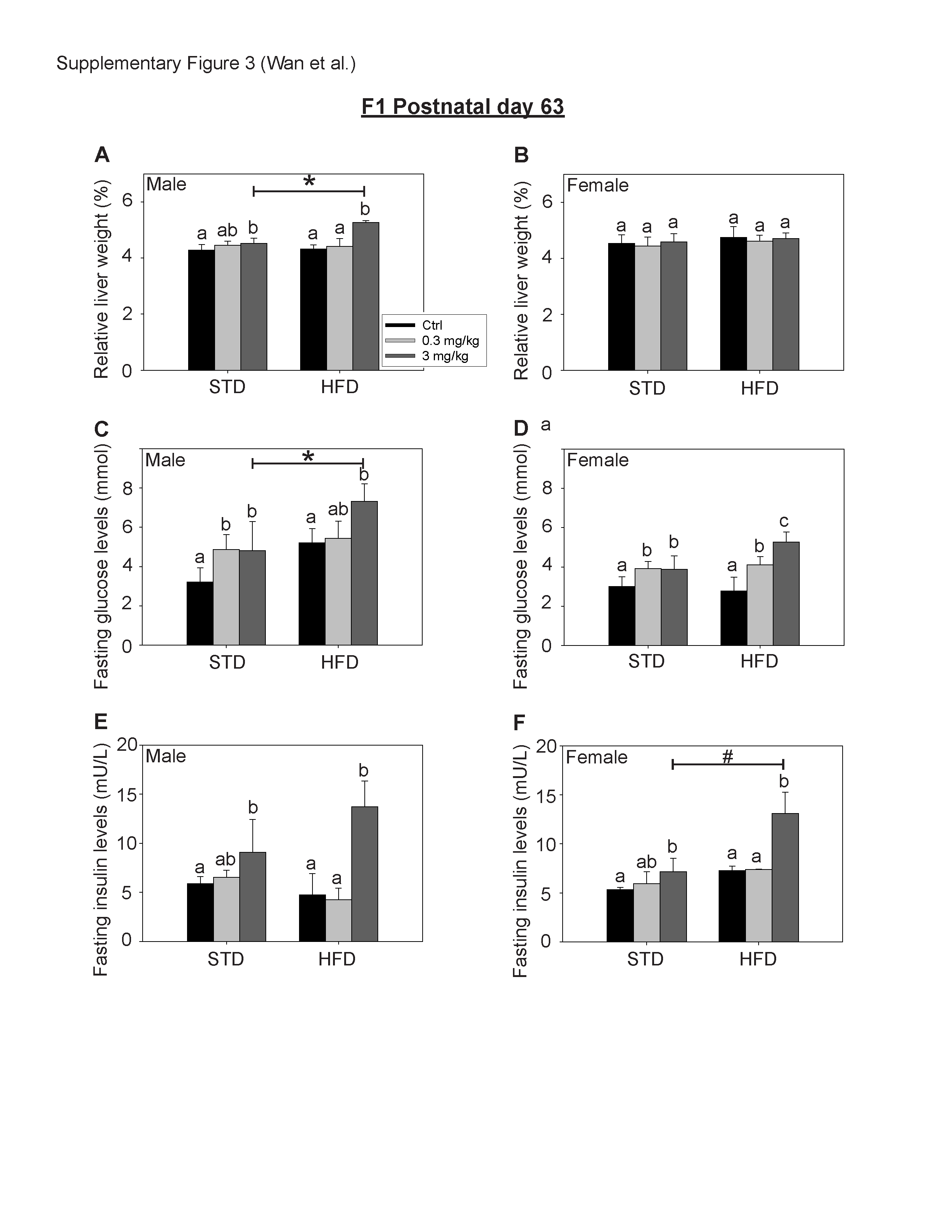

Supplement: Figure S3 — Comparisons of the effects of STD and HFD on perinatal PFOS-exposed F1 adult offspring at PND63. F1 offspring were fed with either standard diet (STD) or high fat diet (HFD) and grown without further PFOS exposure. The relative liver weights, fasting blood glucose and insulin levels of the STD- and HFD-fed F1 were shown. The relative liver weights (n = 8) (A) and fasting blood glucose levels (n = 6) (C) of the HFD-fed male adults (F1) from the high-dose maternal group were significantly higher than that in the respective group in the STD (#p<0.05, student’s t test). (F) Fasting blood insulin levels in the HFD-fed female adults (F1) from the high-dosed exposed group were noticeably increased as compared to the respective group in the STD (n = 6, #p<0.05, Student’s t test). For the comparison of the control and treatment groups under either the STD or HFD, bars with the same letter are not significantly different according to the results of one-way ANOVA followed by Tukey’s test (p<0.05). (TIFF) [file pone.0087137.s003.tiff]

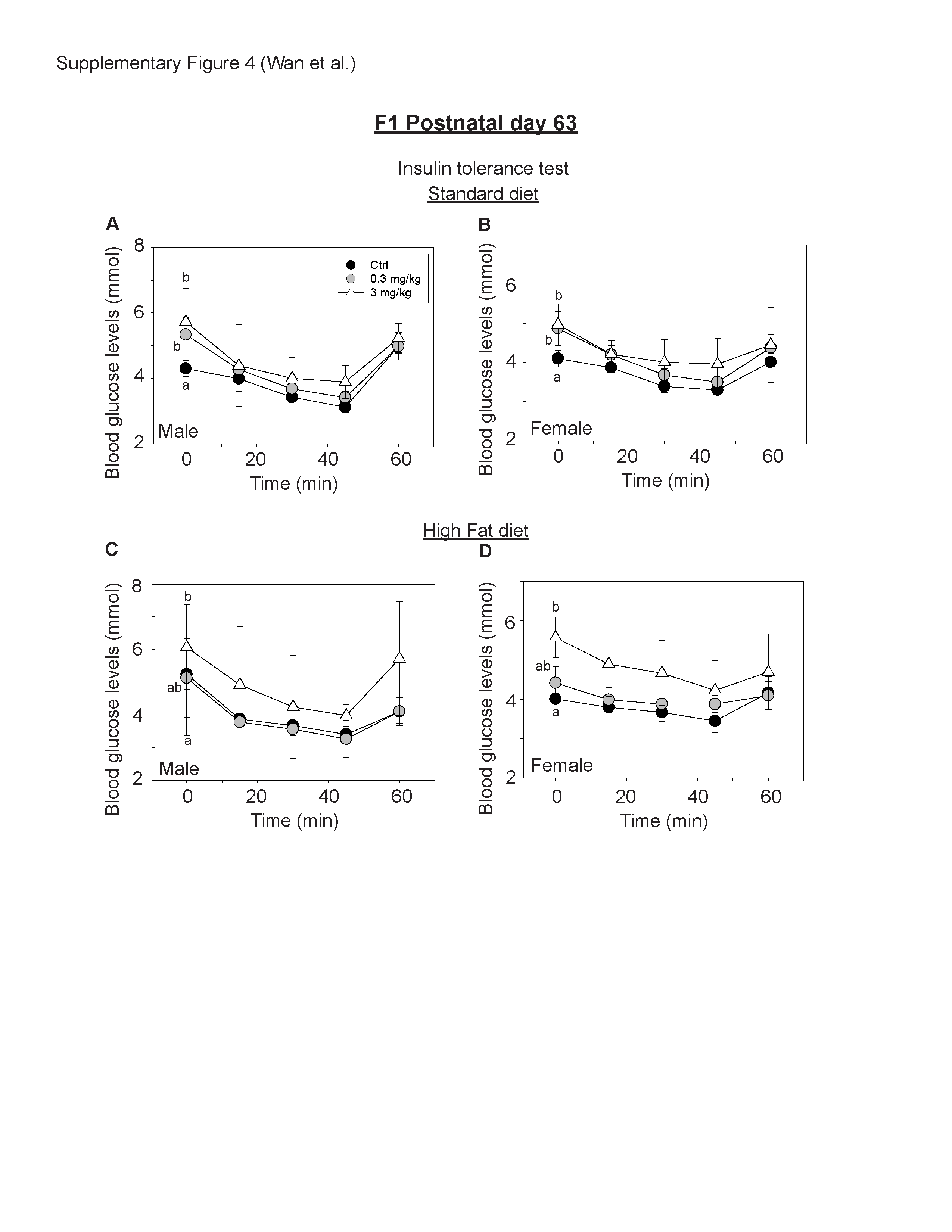

Supplement: Figure S4 — Effects of perinatal PFOS exposure on glucose responses in the insulin tolerance test (ITT). F1 adult offspring were given intraperitoneal injection of insulin (0.5 U insulin/kg body weight) at time 0 (min). Blood glucose levels were measured by the glucometer at the designated time intervals (15, 30, 45 and 60 min). The ITT data of the adult offspring from the STD (A & B) and HFD (C & D) were shown (n = 4). The data at the same time point from the control and the perinatal PFOS treated groups were compared using one-way ANOVA (p<0.05). Statistical analysis showed that there were no significant differences among the control and the perinatal PFOS-exposed groups. (TIFF) [file pone.0087137.s004.tiff]
