# Supplementary material for: Perinatal Exposure to Perfluorooctane Sulfonate Affects Glucose Metabolism in Adult Offspring
Source: PLoS One. 2014 Jan 31;9(1):e87137. doi: 10.1371/journal.pone.0087137 (PMC3909066; doi:10.1371/journal.pone.0087137)
Supplement: Table S1 — Nucleotide sequences of primers used in the present study. (DOC) [file pone.0087137.s005.doc]

**Table S1.** Nucleotide sequences of primers used in the present study.

|  | **Forward** | **Reverse** |
| --- | --- | --- |
| **Fatty acid translocase (*Cd36*)** | GCCAAGCTATTGCGACATGATTA | ATCCGAACACAGCGTAGATAGAC |
| **Cytochrome P450 4A14 (*Cyp4a14*)** | ATTGGTTATGGTTTGCTCCTGTTG | TCATAGTGGAAGGCTGGAGTCA |
| **Insulin-like growth factor-1 (*Igf-1*)** | GCCCCACTGAAGCCTACAAAA | GTACTTCCTTTCCTTCTCCTTTGC |
| **Insulin-like growth factor-1 receptor (*Igf-1r*)** | CAGACACTACTACAAAGGCGT | GATAACGAAGCCATCCGAGTCA |
| **Lipoprotein lipase (*Lpl*)** | ATCAACTGGATGGAGGAGGAGT | TTCTTATTGGTCAGACTTCCTGCT |
| **Insulin receptor (*Ir*)** | CAATGGCAACATCACACACTACC | GCCCTTTGAGACAATAATCCAGC |
| **Prolactin receptor (*Prlr*)** | GGGTCACGGATAACAACATCCT | AACCTTCTTGGCTGATTCCTCAA |
| ***Actin*** | TCTACGAGGGCTATGCTCTCC | TCTTTGATGTCACGCACGATTTC |
